# A Case of Oral Histoplasmosis Concomitant with Pulmonary Tuberculosis

**DOI:** 10.1155/2019/6895481

**Published:** 2019-11-03

**Authors:** Silas Antonio Juvencio de Freitas Filho, Natália Galvão Garcia, Mário César de Souza, Denise Tostes Oliveira

**Affiliations:** ^1^Department of Surgery, Stomatology, Pathology and Radiology (Area of Pathology), Bauru School of Dentistry, University of São Paulo, Bauru, São Paulo, Brazil; ^2^Department of Dentistry, Northern State University of Paraná, Jacarezinho Unit, Paraná, Brazil

## Abstract

The superficial intraoral lesions of histoplasmosis occurring concomitant to tuberculosis, in a 46-year-old man, are reported. The human immunodeficiency virus (HIV) infection test was negative. The immunosuppression caused by tuberculosis in our patient probably had an important role in the development of intraoral lesions of histoplasmosis. Here, we discussed the role of the dentist in the diagnosis of these infectious diseases, highlighting the importance of anamnesis and the histopathology/immunohistochemistry exams.

## 1. Introduction

*H. capsulatum* infects the human host and grows in yeast form [1–7]. The disease may be self-limiting or asymptomatic in healthy individuals, or still to occur in the disseminated form, including the oral cavity [1–7].

In the mouth, the histoplasmosis manifestations may affect any region but are commonly in the tongue, palate, and oropharyngeal mucosa [7, 8]. Furthermore, the oral lesions present from granulomatous nodules to painful shallow or deep ulcers with symptoms of odynophagia and dysphagia [7].

The single oral manifestation of histoplasmosis in immunosuppressed individuals is rare and the diagnosis is challenging [3, 9]. In addition, at the time of diagnosis of oral histoplasmosis, the health professional should investigate the presence of concomitant diseases, such as malignant neoplasms or other infections as tuberculosis [10].

The occurrence of oral histoplasmosis in patients with pulmonary tuberculosis has been reported in some studies mainly due to immunosuppression and physical weakness caused by bacterial disease [8, 10, 11]. The tuberculosis has been concomitantly diagnosed in approximately 10% of Brazilians with histoplasmosis [12]. Antonello et al. [8] showed that 36% of patients with oral histoplasmosis had concomitant active pulmonary tuberculosis, 18% had malignant neoplasia, 9% had chronic obstructive pulmonary disease, and 9% had no other disease at the time of diagnosis of fungal infection.

Here, we report a case of oral histoplasmosis in a patient with a diagnosis of pulmonary tuberculosis. The role of the dentist in the diagnosis of this infectious disease including the importance of detailed anamnesis and the histopathology/immunohistochemistry exams is discussed.

## 2. Case Report

**Figure 1:**
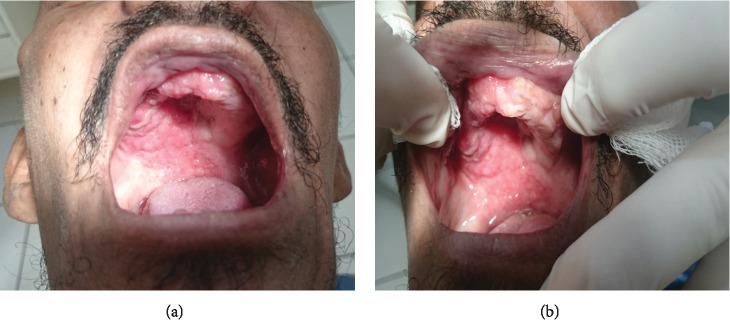
Clinical aspect of intraoral lesions in the palate and alveolar ridge regions (a, b).

A 46-year-old man was attended in the dental clinic complaining of symptomatic oral lesions with two months in duration. The intraoral physical examination revealed diffuse, friable, vegetative areas on the right upper alveolar ridge, hard palate, and left inferior alveolar ridge (Figures 1(a) and 1(b)). His medical history revealed a diagnosis of tuberculosis about a month ago in which the expectorated sputum smears were positive for bacteria and acid-fast bacilli. In addition, at the time of diagnosis of tuberculosis, the patient had a significant weight loss and asthenia. The patient was under antibacterial therapy (oral isoniazid (INH) 225 mg/day, rifampicin (RFP) 450 mg/day, pyrazinamide 1,200 mg/day, and ethambutol (EB) 825 mg/day). Testing for human immunodeficiency virus (HIV) infection was negative. Furthermore, the patient confirmed smoking and chronic alcoholism. He worked as a night flow controller on the side of a highway and lived very close to the countryside. After knowing the patient's medical history, the main hypothesis for oral lesions was tuberculosis.

**Figure 2:**
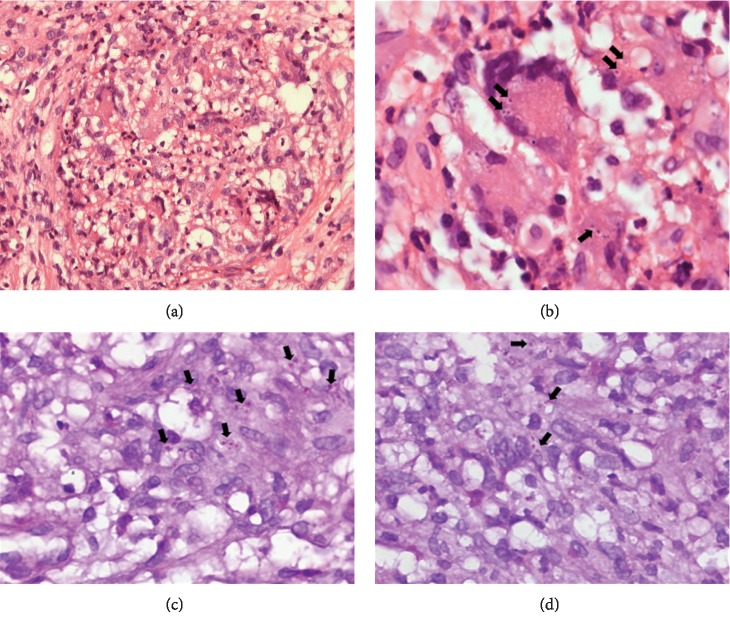
Connective tissue with intense inflammatory infiltrate with a granulomatous pattern, consisting of giant multinucleated inflammatory cells and vacuolated macrophages, with several fungi suggestive of *H. capsulatum—*(hematoxylin-eosin stain; (a) ×200, (b) ×400). In (c) and (d), the periodic acid-Schiff (PAS) staining showed vacuolated macrophage with positivity for *H. capsulatum* ((c d) ×400). Note the numerous small rosy dots (arrow).

An incisional biopsy of the right upper alveolar ridge showed connective tissue with intense inflammatory infiltrate with a granulomatous pattern, consisting of giant multinucleated inflammatory cells and vacuolated macrophages, with innumerable fungi suggestive of *H. capsulatum* (Figures 2(a) and 2(b)). Staining slides with periodic acid-Schiff (PAS) (Figures 2(c) and 2(d)) and Grocott-Gomori methenamine silver were positive for the morphological characteristics of *H. capsulatum*. In addition, the immunohistochemical reactivity to Histoplasma using polyclonal antibody was positive; for polyclonal *P. brasiliensis, Leishmania spp.* and Calmette-Guérin bacillus were negative. The diagnosis of oral histoplasmosis was established. We did not search for fungi in other biological samples.

**Figure 3:**
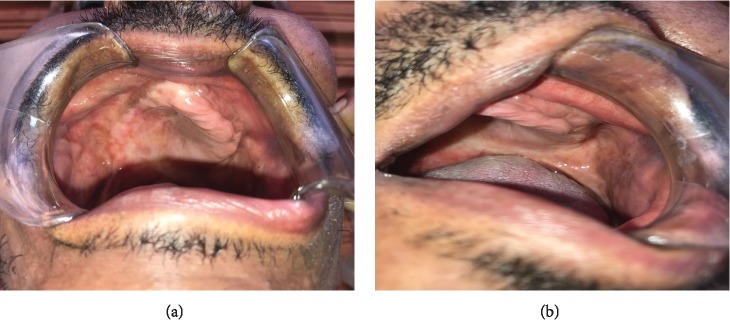
After twelve months, the clinical regression of oral histoplasmosis lesions.

Initially, the drug was maintained for tuberculosis and prescribed fluconazol (400 mg/day) for seven months for treatment of oral histoplasmosis. During the follow-up, when a gradual increase in body weight was noted, fluconazole was substituted for itraconazole 200 mg/day for eight months with the resolution of oral histoplasmosis lesions. The clinical control one year after initiation of itraconazole treatment can be seen in Figures 3(a) and 3(b). One year after the initial treatment of tuberculosis, the patient was cured.

## 3. Discussion

Tuberculosis remains a public health problem in many countries including Brazil; and with the immunosuppression resulting from the disease, some opportunistic infections may develop, especially in cases associated positive HIV [10, 13]. In the present case reported, a 46-year-old man who presented to her dentist with superficial lesions located in several intraoral sites was in treatment for tuberculosis. The detailed clinical investigation showed that our patient was HIV negative and the oral histoplasmosis diagnosis was established after laboratory exams excluding other infections. The immunosuppression caused by tuberculosis in our patient probably had an important role in the development of intraoral lesions of histoplasmosis.

Sometimes, the diagnosis of intraoral histoplasmosis is challenging because the lesions can be mimicking malignancies, other fungal diseases, or traumatic ulcers, and the biopsy has been a useful resource to establish the final diagnosis [1, 2, 14]. In routine staining (hematoxylin and eosin), PAS and Grocott-Gomori silver methylamine staining can identify fungi within prominent macrophages and giant Langhans-type giant cells. In summary, initially, the histopathology directed our diagnostic hypotheses and, finally, the use of immunohistochemistry was essential to eliminate other oral infectious diseases and establish the final diagnosis. Although we used polyclonal antibodies in our pathological investigation, the analysis made it possible to eliminate the possibility of other infections, including oral tuberculosis and paracoccidioidomycosis. Besides, serology and culture tests may assist in establishing the diagnosis of this fungal disease [1].

Tuberculosis mainly affects the lungs and can present several complications in its clinical course causing weakness, cough, weight loss, shortness of breath, among other signs and symptoms, and the possibility of concomitant infections [1, 10]. As shown in our case, weight loss and asthenia are common clinical signs in patients with concomitant active pulmonary tuberculosis and histoplasmosis [8]. In addition, dysphagia and fever can also be found among these patients [8]. Interestingly, at the time of diagnosis, our patient had several complications but the laboratory tests were negative for HIV. In case a similar to ours, the main suspect has been of oral tuberculosis [2]. All these clinical characteristics and information collected during the anamnesis become the diagnostic process challenging.

Initially, our patient could not be treated with itraconazole. The physician instituted this medicament after effective response to tuberculosis treatment. This decision was also made due to the drug interaction between rifampicin and itraconazole, where itraconazole levels significantly decrease in the presence of the other, so these two drugs should not be administered concomitantly [15, 16]. Additionally, amphotericin B deoxycholate is another medicament that can be used to treat acute and chronic cavitary pulmonary histoplasmosis [17].

*H. capsulatum* has been considered a fungus endemic in the Mississippi and Ohio River Valleys, also in Central and South America, Asia, and Australia [3, 6, 7, 14]. In all Brazilian regions, this infectious disease has been very common in men between the fourth and fifth decades of life with a high mortality rate; this data may be underestimated due to the lack of mandatory reporting [12]. Moreover, in Brazil, oral lesions of histoplasmosis may lead the dentist to suspect of other infections such as the paracoccidioidomycosis [3, 7].

Although the number of fungal oral lesions diagnosed in Brazilian Referral Centers is relatively low [7, 18], the cases of disseminated histoplasmosis with oral manifestation have increased in recent years, especially in South American men [1] and consequently has caused concern. Then, the biopsy for histopathology and culture of oral suspected lesions by dentists, particularly in immunosuppressed patients, is mandatory for establishing the diagnosis of this fungal infection.

## 4. Conclusion

In summary, it is prudent for the dentist to investigate the patient's health status considering the opportunistic oral mucosal infections, especially in immunosuppressed patients. The clinical diagnosis of oral histoplasmosis can be challenging, and a detailed anamnesis associated with complementary laboratory tests are required. Correct therapeutic indication and prolonged follow-up are essential for patient healing to avoid recurrence of this fungal infection.

## References

[1] Pincelli T., Enzler M., Davis M., Tande A. J., Comfere N., Bruce A. (2019). Oropharyngeal histoplasmosis: a report of 10 cases. *Clinical and Experimental Dermatololy*.

[2] Chatterjee D., Chatterjee A., Agarwal M. (2017). Disseminated histoplasmosis with oral manifestation in an immunocompetent patient. *Case Reports in Dentistry*.

[3] Klein I. P., Martins M. A., Martins M. D., Carrard V. C. (2016). Diagnosis of HIV infection on the basis of histoplasmosis‐related oral ulceration. *Special Care in Dentistry*.

[4] Brazão-Silva M. T., Mancusi G. W., Bazzoun F. V., Ishisaki G. Y., Marcucci M. (2013). A gingival manifestation of histoplasmosis leading diagnosis. *Contemporary Clinical Dentistry*.

[5] Vidyanath S., Shameena P., Sudha S., Nair R. G. (2013). Disseminated histoplasmosis with oral and cutaneous manifestations. *Journal of Oral and Maxillofacial Pathology*.

[6] Sharma D., McKendry A., Nageshwaran S., Cartledge J. (2012). A case of oral ulceration and disseminated histoplasmosis in HIV infection. *International Journal of STD & AIDS*.

[7] Ferreira O. G., Cardoso S. V., Borges A. S., Ferreira M. S., Loyola A. M. (2002). Oral histoplasmosis in Brazil. *Oral Surgery, Oral Medicine, Oral Pathology, Oral Radiology, and Endodontology*.

[8] Antonello V. S., Zaltron V. F., Vial M., Oliveira F. M., Severo L. C. (2011). Oropharyngeal histoplasmosis: report of eleven cases and review of the literature. *Revista da Sociedade Brasileira de Medicina Tropical*.

[9] Fortuna G., Mignogna M. D. (2011). Oral histoplasmosis of a healthy man in a non-endemic area. *Infection*.

[10] Cui Z., Lin M., Nie S., Lan R. (2017). Risk factors associated with tuberculosis (TB) among people living with HIV/AIDS: a pair-matched case-control study in Guangxi, China. *PLoS One*.

[11] Nabet C., Belzunce C., Blanchet D. (2018). *Histoplasma capsulatum* causing sinusitis: a case report in French Guiana and review of the literature. *BMC Infectious Diseases*.

[12] Almeida M. A., Almeida-Silva F., Guimarães A. J., Almeida-Paes R., Zancopé-Oliveira R. M. (2019). The occurrence of histoplasmosis in Brazil: a systematic review. *International Journal of Infectious Diseases*.

[13] Kritski A., Andrade K. B., Galliez R. M. (2018). Tuberculosis: renewed challenge in Brazil. *Revista da Sociedade Brasileira de Medicina Tropical*.

[14] Hendren N., Yek C., Mull J., Cutrell J. B. (2017). Disseminated histoplasmosis presenting as multiple oral ulcers. *BMJ Case Reports*.

[15] Moon S. M., Park H. Y., Jeong B. H., Jeon K., Lee S. Y., Koh W. J. (2015). Effect of rifampin and rifabutin on serum itraconazole levels in patients with chronic pulmonary aspergillosis and coexisting nontuberculous mycobacterial infection. *Antimicrobial Agents and Chemotherapy*.

[16] Blomley M., Teare E. L., de Belder A., Thway Y., Weston M. (1990). Itraconazole and anti-tuberculosis drugs. *The Lancet*.

[17] Mahajan V. K., Raina R. K., Singh S. (2017). Case report: histoplasmosis in Himachal Pradesh (India): an emerging endemic focus. *The American Journal of Tropical Medicine and Hygiene*.

[18] Vasconcelos A. C., Aburad C., Lima I. F. P. (2017). A scientific survey on 1550 cases of oral lesions diagnosed in a Brazilian referral center. *Anais da Academica Brasileira de Ciências*.

